# Synthesis, Characterization, Acetylcholinesterase Inhibition, Molecular Modeling and Antioxidant Activities of Some Novel Schiff Bases Derived from 1-(2-Ketoiminoethyl)piperazines

**DOI:** 10.3390/molecules16119316

**Published:** 2011-11-07

**Authors:** Saleh M. Salga, Hapipah M. Ali, Mahmood A. Abdullah, Siddig I. Abdelwahab, Lam Kok Wai, Michael J. C. Buckle, Sri Devi Sukumaran, A. Hamid A. Hadi

**Affiliations:** 1 Department of Chemistry, University of Malaya, Kuala Lumpur, 50603, Malaysia; 2 Department of Molecular Medicine, University of Malaya, Kuala Lumpur, 50603, Malaysia; 3 Department of Pharmacy, University of Malaya, Kuala Lumpur, 50603, Malaysia; 4 Faculty of Pharmacy, Universiti Kebangsaan Malaysia, Jalan Raja Muda Abdul Aziz, Kuala Lumpur, 50300, Malaysia

**Keywords:** 1-(2-ketoiminoethyl)piperazines, AChE inhibition, molecular docking, antioxidant study, acute toxicity

## Abstract

Some novel Schiff bases derived from 1-(2-ketoiminoethyl)piperazines were synthesized and characterized by mass spectroscopy, FTIR, UV-Visible, ^1^H and ^13^C-NMR. The compounds were tested for inhibitory activities on human acetylcholinesterase (hAChE), antioxidant activities, acute oral toxicity and further studied by molecular modeling techniques. The study identified the compound (DHP) to have the highest activity among the series in hAChE inhibition and DPPH assay while the compound LP revealed the highest activity in the FRAP assay. The hAChE inhibitory activity of DHP is comparable with that of propidium, a known AChE inhibitor. This high activity of DHP was checked by molecular modeling which showed that DHP could not be considered as a bivalent ligand due to its incapability to occupy the esteratic site (ES) region of the 3D crystal structure of hAChE. The antioxidant study unveiled varying results in 1,1-diphenyl-1-picrylhydrazyl (DPPH) and ferric reducing antioxidant power (FRAP) assays. This indicates mechanistic variations of the compounds in the two assays. The potential therapeutic applications and safety of these compounds were suggested for use as human acetylcholinesterase inhibitors and antioxidants.

## 1. Introduction

**Figure 1:**
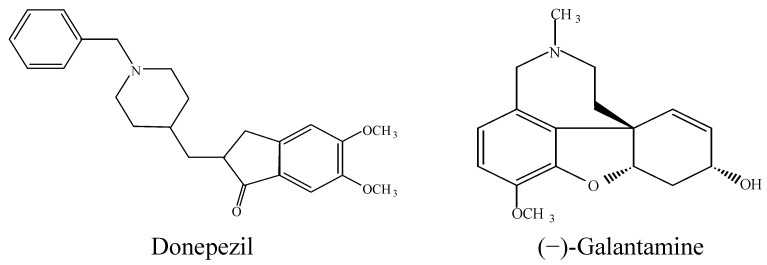

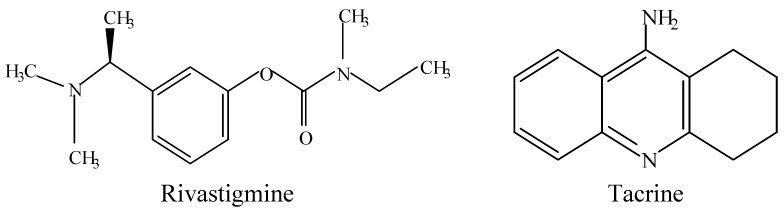
Acetylcholinesterase inhibitors.

The neurodegenerative disorder characterized by the nerve cell dysfunctions and loss of neurons in the central nervous system was first discovered in 1907 by a German scientist, Alois Alzheimer, and was named as Alzheimer’s disease (AD). Millions of people are reported to fall victims of this traumatic problem worldwide [1]. At the early stages, the patient is faced with a decline in cognitive functions, exclusively short-term memory, which later resulta in the incapability to read, speak and/or think rationally [2]. Recent reports on curative approaches to this ailing disease are based on the assumption of a cholinergic mechanism, with particular emphasis on acetylcholinesterase inhibition [2,3]. Indeed, many scientific trials have been conducted in order to discover typical and non-toxic drug for the treatment of AD. The most commonly used drug for the treatment of AD is tacrine which is recognized to have so many side effects that in most cases they lead to withdrawal of the medication. Other drugs like donepezil, rivastigmine, galanthamine, caproctamine and memantine were also used for the treatment of AD, but are known for their unfavorable effects like hepatotoxicity, gastrointestinal disturbance, dizziness, diarrhea, vomiting and nausea [4,5,6]. The key factor liley implicated in many diseases is oxidative stress caused by free radicals produced as a result of an imbalance in antioxidants produced by the cells [7]. This suggests that treatment of AD should involve acetylcholinesterase inhibitors and antioxidants that can scavenge the excess free radicals and antagonize the consequences of oxidative stress [8,9]. In addition, the literature reports that many standard drugs contain a polyamine backbone in their structures (Figure 1), and that the AChE gorge is lined with various preserved aromatic residues which in principle can form cation-π bonds with a basic polyamine counterpart [10,11]. This study therefore sought to synthesize and characterize some new compounds capable of AChE binding site inhibition and compares their activity with that of standard drugs like tacrin and propidium. These compounds have flexidentate behavior and contain secondary and tertiary amines in their structures akin to the secondary amino function found in tacrine dimer [12] and the tertiary amino group of donepezil [13]. Moreover, the compounds were tested for acute toxicity and antioxidant activities using the FRAP and DPPH assays due to arising demands for compounds that are safe and possess dual AChE inhibition and antioxidant activities [14,15].

## 2. Results and Discussion

### 2.1. Chemistry

**Scheme 1:**
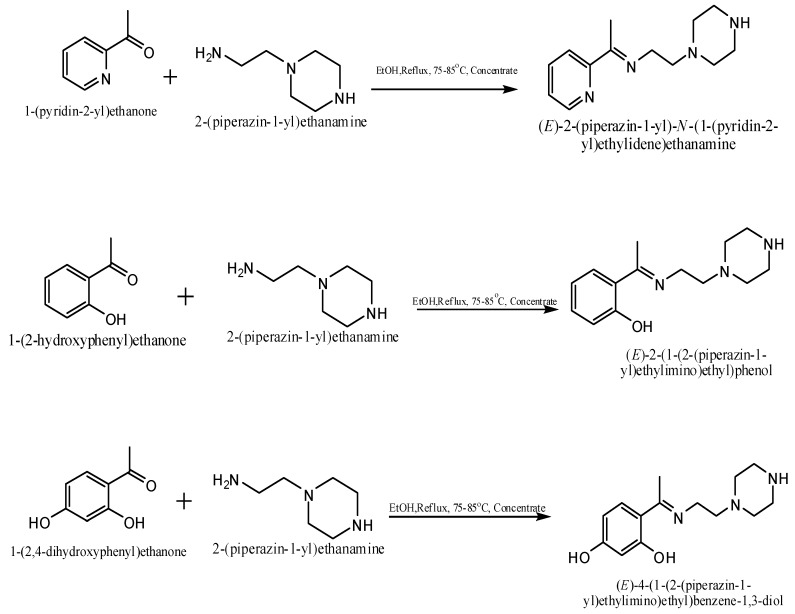
Reaction pathway.

The reaction of 2-(piperazin-1-yl)ethanamine with selected ketones resulted in the formation of the corresponding 1-(2-ketoiminoethyl)piperazines that exhibited NMR, IR and UV-Visible spectra consistent with the proposed structures that allowed the synthesized compounds to be identified as 2-(piperazin-1-yl)-*N*-(1-(pyridin-2-yl)ethylidene)ethanamine (**LP**), 2-(1-(2-piperazin-1-yl)ethylimino) ethyl)phenol (**2HP**) and 4-(1-(2-piperazin-1-yl)ethylimino)ethyl)benzene-1,3-diol (**DHP**), respectively (Scheme 1).

The IR spectra of all the compounds revealed complete absence of the characteristic ketonic carbonyl and displayed absorptions at wavelengths of 1,654, 1,616, and 1,595 cm^−1^ for **LP**, **2HP** and **DHP**, respectively, which correspond to the characteristic iminic frequency [16,17], thus, confirming the formation of Schiff bases. The broad bands observed at 3,394 cm^−1^ in the spectra of **2HP** and **DHP** could be attributed to the phenolic intra-molecular hydrogen bonding of their O–H groups [18]. Other series of bands observed in the spectra of compounds **LP**, **2HP** and **DHP** in the wavelength ranges {2,823, 2,821, 2,833 cm^−1^}, {1,459, 1,302, 1,243 cm^−1^} {1,152, 1,152, 1,137cm^−1^} can be assigned to υ(C-H) aliphatic, υ(C-C) aromatic and υ(C-N) stretching vibrations, respectively [19,20].

**Figure 2:**
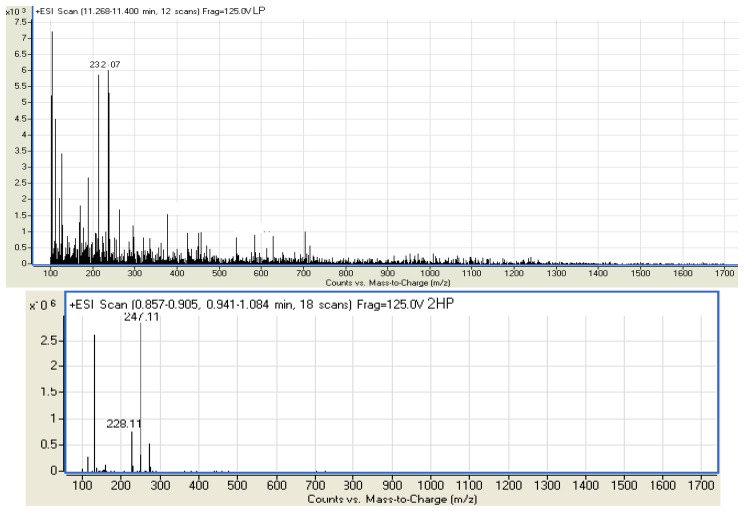

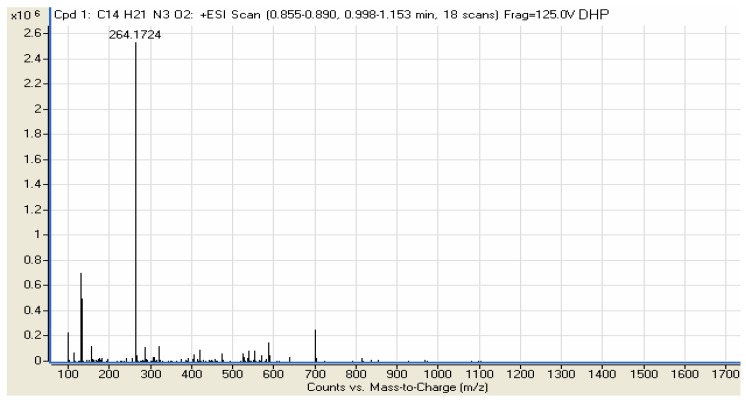
Mass spectra for the compounds **LP**, **2HP** and **DHP**.

The proton NMR spectra of the Schiff bases revealed a singlet δ 1.13–2.01 which is due to a methyl group shielded by the imine moiety. The compound **LP** did not show the presence of OH protons which are observed in the spectra of **2HP** and **DHP** at δ 6.74 ppm and δ 6.17 ppm, respectively, and instead it shows triplet at δ.7.43–7.84. In the ^13^C-NMR spectra of the compounds **LP**, **2HP** and **DHP** the methylene group peak overlaps with DMSO solvent peak at δ 39–46, while the aromatic carbons were observed at around δ 120 in all the spectra. In addition the compounds gave M+H ion peaks in the mass spectra. The liquid chromatography electron spray ionization mass spectrum (LC-ESI-MS) of compound **LP** gave a molecular ion peak of 232.07 Da which correlates to the calculated weight of C_13_H_20_N_4_ (232.17), thus proving the purity of the compound. The compound **2HP** also shows a molecular ion peak at 247.11 Da, also very close to the calculated weight of C_14_H_21_N_3_O (247.17). Finally, the mass spectrum of **DHP** gave a [M+H] molecular ion peak of 264.17 Da whereby the calculated mass for C_14_H_21_N_3_O_2_ is 263.16. This is in complete agreement with the proposed structure and also proves the purity of the compound (Figure 2). IR and UV-Visible spectral data were also all in accordance with the assigned structures The UV-visible spectra of the compounds LP, 2HP and DHP shows absorption bands at 284, 242 and 215 nm respectively, which may be assigned to benzene ring π→π* transitions [21,22,23,24,25].

### 2.2. Anti-AChE Assay

The inhibitory activities of the compounds **LP**, **2HP** and **DHP** on human acetylcholinesterase were 9.9, −4.0, and 21.8% respectively (Table 1). The activities were only fair when compared to the standard drugs tacrin and propidium used in this study which showed inhibitions of 76.6% and 28.2%, respectively. The results indicated that the compound DHP had the highest activity which can be ascribed to the increased resonance effect caused by the hydroxyl group of the aromatic ring.

### 2.3. Molecular Docking

**Figure 3:**
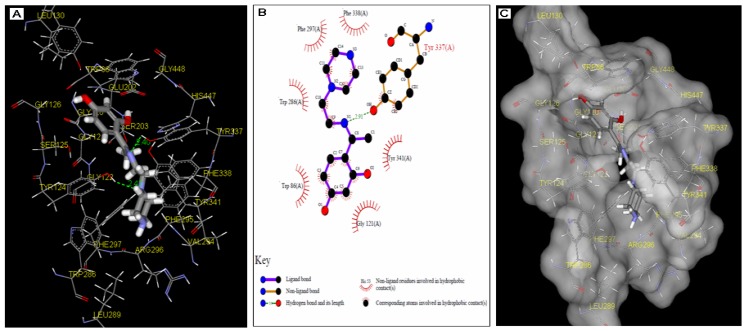
A close view of the potential interaction between DHP and the binding site of hAChE (pdb id: 1B41). **A**: Binding interactions of DHP in the catalytic site of TcAChE (green stick = compound **5**). The most important residues involved in the interactions with DHP including the peripheral anionic site (PAS) and anionic subsite (choline-binding subsite); **B**: The 2D representation docking result of DHP with the residues in the active site of hAChE; **C**: Interatomic contacts (yellow) of the ligand with the residues in the active site of hAChE.

The crystal structure of hAChE (pdb id: 1B41) shows that this enzyme possesses a deep narrow gorge which penetrates halfway into the enzyme, where the catalytic site resides [26]. Conversely, hAChE shares a certain degree of similarity with both mAChE and TcAChE, especially the active-site gorge due to their high degree of sequence identities of 87% and 53%, respectively. Generally, the catalytic site of AChE consists of five regions, which are the peripheral anionic site (PAS), the acyl pocket, the esteratic site (ES), the oxyanion hole (OH) and the anion subsite (AS). A similar catalytic site is also observed in hAChE, PAS (Tyr 72, Tyr 124, Tyr 341, Asp 74 and Trp 286 residues), the acyl pocket (Phe 297, Phe 295 and Phe 338 residues), CT (Ser 203, His 447 and Glu 202 residues), OH (Gly 121, Gly 122 and Ala 204 residues) and AS (Trp 86 and Glu 202 residues). A molecular docking simulation (Figure 3) showed that **DHP** is well positioned at the active-site gorge. The hydrophobic interaction between **DHP** (the piperazine and the alkyl linker) and the rich aromatic contents (Tyr 124, Tyr 341, Tyr 337, Phe 338, Phe 297 and Trp 286) along the gorge could potentially direct the phenyl ring to penetrate deep into the anionic site and oxyanion hole regions in the choline-binding site. This interaction is further intensified, where three hydrogen bonding were found between **DHP** and the residues in the active site. This includes hydrogen bonding between the tertiary nitrogen of the piperazine ring with Tyr 124 residue at a distance of 2.42 Å. Two hydrogen bonding interactions between the imine group and two amino residues along the gorge which are Tyr 124 (3.05 Å) and Tyr 337 (2.91 Å) were also observed during the docking experiment (Figures 3A and 3C).

These interactions could possibly help to dock the phenyl moiety to the anionic site. We believe that apart from the hydrophobic interactions, these hydrogen bonding effects are crucial for the AChE inhibition activity shown by **DHP**. It was clearly observed that **DHP** exhibited much higher AChE inhibition activity in comparison with **LP** and **2H**P. It becomes visibly clear that the *para*-hydroxyl group functionality plays an important role in the inhibition activity. Close inspection of the docking results revealed that the *para*-hydroxyl group is in close proximity (within the range of 3.13 Å) to the hydroxyl of the Tyr 124 side chain. This interaction could possibly aid the phenyl ring of DHP to engage in a π-π stacking interaction with the indole ring of Trp 86 (Figure 3B). It is known that inhibitors such as acridine exhibit such interactions with the conserved residue of Trp 86 [27]. Overall, the binding of DHP is energetically favorable, with a predicted –CDOCKER energy of 19.148 and –CDOCKER interaction energy of 40.816.

### 2.4. Antioxidant Assays

The antioxidant efficacies of the Schiff bases **LP**, **2HP**, and **DHP** were tested and the results obtained revealed different activities in the two assays. This indicates that two mechanisms operating in different ways must be responsible for the observed activity. The color change from deep purple to yellow at 515 nm observed in the DPPH assay has confirmed the radical scavenging activity of the compounds. A reference curve of absorbance (A) against DPPH concentration in methanol was plotted and used for the calculation of DPPH concentration at various reaction times (R^2^ = 0.9999). Only the compound **DHP** shows IC_50_ values of 25 µg/mL among the series (Table 1). The positive control used (ascorbic acid) showed an IC_50_ value of 1.4 ± 0.71 µg/mL. The high activity of the compound **DHP** in the DPPH assay can be related to the resonance effect of the hydroxyl at the *meta*-position to the phenolic group in the compound, whereupon electron donating substituents increase the electron density into the aromatic ring making it more reactive towards electrophilic attack. This presumably promotes the release of phenolic hydrogen to the (1,1-diphenyl-2-picrylhydrazyl) free radical indicated by a color change from purple to yellow.

The second method used for testing the antioxidant activities of these compounds was the FRAP assay. It is considered an accurate method for assessing “antioxidant power”. Ferric to ferrous ion reduction at low pH causes a colored ferrous-tripyridyltriazine complex to form. FRAP values are obtained by comparing the absorbance change at 593 nm in test reaction mixtures with those containing ferrous ions at known concentrations. In this study, the compounds **LP**, **2HP** and **DHP** showed FRAP assay values of 1,464.7, 293.3, and 355.3, respectively (Table 1) which is above the value of 187.3 shown by BHT, but below the value of 1,552.7 observed for ascorbic acid used as standards. It was observed that **DHP** demonstrates the highest potential activities in the DPPH assay while **LP** shows the highest in FRAP assay. The high activity of **LP** can seemingly be due to increased in π-electron delocalization within the pyridine ring which increases the electron density and causes ferric ion to change to ferrous [28].

### 2.5. Acute Toxicity

Oral administration of 1-(2-ketoiminoethyl)piperazines at doses lower than 5 g/kg causes no apparent toxicity. However, above 5 g/kg the 1-(2-ketoiminoethyl)piperazines slowed animal movement, decreased aggressiveness, and touch and pain sensibility were noticed. The animals did not show gross negative behavioral changes such as excitement, respiratory distress, convulsions or coma. All the observed signs appeared 2–3 h after 1-(2-ketoiminoethyl)piperazine administration and disappeared dose-dependently. No mortality was observed up to 14 days. The biochemical data showed no significant change in serum albumin, liver enzymes (AST, ALT and ALP), total protein content, globulins, conjugated and total bilurubin. Blood concentrations of creatinine, urea, sodium, potassium, chloride carbon dioxide and anion gap were analyzed. All the parameters checked were found within the expected range in the animals treated with the compounds (*P <* 0.05–0.001) when compared to the normal (control) group (Table 2 and Table 3).

## 3. Experimental

### 3.1. General

The compounds synthesized in this study were characterized by spectral methods. Mass spectra were determined using a ABI 4800 Maldi TOF/TOF mass spectrophotometer (BIDMC Genomics, Proteomics And Bioinformatics Core, Boston, MA, USA) (LC-MS, ESI, 125.0 V), IR spectra was recorded at the wavelength range from 4,000–400 cm^−1^ using a Perkin Elmer 783 spectrophotometer, NMR spectra were obtained on a ECA400 FT-NMR spectrophotometer using TMS as internal standard, UV-visible spectra were recorded on a UV-1650PC model UV-visible spectrophotometer.

### 3.2. 2-(Piperazin-1-yl)-N-(1-(pyridin-2-yl) ethylidene)ethanamine *(**LP**)*

A stoichiometric amount of 2-(piperazin-1-yl)ethanamine (0.13 g, 1 mmol) in an absolute ethanol (25 mL) was refluxed with 1-(pyridine-2-yl)ethanone (0.12 g, 1 mmol) also dissolved in ethanol (25 mL) at a temperature of 75–85 °C for 3 h, to give an orange solution which after evaporation gave a brownish-red oil. The oily product produced a hygroscopic solid after three days. This was dissolved in methanol and heated to 55–60 °C with addition of a few drops of NaClO_4_ (0.1 g) taken in methanol (1 mL). The product, after evaporating the solvent under reduced pressure, formed a brown solid. Recrystallization from a 70:30 methanol/water mixture was performed to give 0.098 g of the title compound (42.2% yield). IR (KBr, cm^−1^) 3,413 s υ (N-H), 2,838 s υ (C-H), 1,633 s υ (C=N), 1,271 s υ (C-C), 1,134 s, υ (C-N).^1^H-NMR (DMSO-*d*6), δ, ppm: 1.77 (s, 3H, CH_3_-iminic), 2.24 (s, 1H, NH), 2.43 (d, 4H, 2CH_2_N), 2.63 (t, 4H, 2CH_2_NH), 7.43 (t, 1H, arH, CHCHN), 7.84 (t, 1H, arH, CHCHCHN), 8.04 (d, 1H, arH, CHCHCHCHN), 8.60 (d, 1H, pyridinic). ^13^C-NMR (DMSO-*d*6), δ, ppm: 14.12 (CH3), 29.24 (CH2), 39.99–46.10 (DMSO-*d*6 + CH2), 49.12 (CH2), arC: [121.65 (CH), 124.91 (CH), 136.97 (CH), 149.72 (CH), 166.8 (CH)]; UV: 284 nm (3075.25 mol^−1^Lcm^−1^), 351 nm (3313.43 mol^−1^Lcm^−1^). MS: calc. *m/z* 232.17, found 232.07.

### 3.3. 2-(1-(2-Piperazin-1-yl)ethylimino)ethyl)phenol *(**2HP**)*

An ethanolic solution (25 mL) containing 2-(piperazin-1-yl)ethanamine (0.13 g, 1 mmol) was refluxed with 1-(2-hydroxyphenyl)ethanone (0.14 g, 1 mmol) in ethanol (25 mL) for 3 h at a temperature of 75–85 °C. A golden yellow solution was formed which after evaporating the solvent produced a yellow oil. This produces a hygroscopic solid after five days under vacuum. The solid product was dissolved in methanol at 55–60 °C. Solid NaClO_4_ (0.1 g) was added to the solution while it was hot and the suspension was immediately filtered. The filtrate was concentrated in air to give a needle-like microcrystalline solid after three days. The yellowish crystals were collected by filtration, washed with diethyl ether and recrystallized from a hexane-dichloromethane mixture (70:30), but the quality of the crystals was not suitable for x-ray diffraction. Yield: 0.124 g (50.2%). IR (KBr, cm^−1^) 3,394 s υ (N-H), 2,821 s υ (C-H), 1,616 s υ (C=N), 1,302 s υ (C-C), 1,152 s υ (C-N). ^1^H-NMR (DMSO-*d*6), δ, ppm: 1.13 (s, 3H, CH_3_-iminic), 2.38 (s, 1H, NH), 2.57 (d, 4H, 2CH_2_N), 2.60 (t, 4H, 2CH_2_NH), 6.78 (m, arH, CHCHCHCHCOH), 7.26 (m, arH, CHCHCOH), 7.28 (m, 1H, arH, CHCHCHCOH), 7.59 (m, arH, CHCHCOH), 7.61 (d, 1H, phenolic) ^13^C-NMR (DMSO-*d*6), δ, ppm: 19.03 (CH3), 28.15 (CH2), 39.73–46.52 (DMSO-*d*6 + CH2), 54.80–59.08 (CH2), arC: [116.78 (CH), 118.88 (CH), 131.79 (CH), 132.84 (CH), 164.88 (CH), 172.92 (CH)]; UV: 262 nm (30490.97 mol^−1^Lcm^−1^), 318 nm (14590.03 mol^−1^Lcm^−1^). MS: calc. *m/z* 247.17, found 244.97 (M + H).

### 3.4. 4-(1-(2-Piperazin-1-yl)ethylimino)ethyl)benzene-1,3-diol *(**DHP**)*

A weighed amount of 2-(piperazin-1-yl)ethanamine (0.13 g, 1 mmol) in ethanol (25 mL) was mixed with a stirred ethanolic solution (25 mL) of 1-(2,4-dihydroxyphenyl)ethanone (0.15 g, 1 mmol) at room temperature and refluxed for 3 h to give a clear orange solution. This was evaporated a using rotary evaporator to obtain an orange oil which after one week produces a white hygroscopic solid. The solid product was dissolved in methanol and heated to 55–60 °C. Solid NaClO_4_ (0.1 g) was added to the solution while hot and the suspension was filtered instantly. The filtrate produces a white solid after cooling. which was filtered off, washed with an ethanol-water mixture and dried under vacuum. Yield: 0.132 g (50.2%). IR (KBr, cm^−1^) 3,394 s υ (N-H), 2,833 s υ (C-H), 1,595 s υ (C=N), 1,243 s υ (C-C), 1,137 s υ (C-N). ^1^H-NMR (DMSO-*d*6), δ, ppm: 2.01 (s, 3H, CH_3_-iminic), 2.32 (s, 1H, NH) 2.64 (d, 4H, 2CH_2_N), 3.55 (t, 4H, 2CH_2_NH) 6.15 (t, arH, CHCOHCHCOH), 7.31 (s, 1H, arH, OHCCHCOH), 7.33 (d, arH, CHCHCOHCHCOH), 7.53 (s, 1H, arH, OHCCHCOH), 7.55 (s, 1H, phenolic) ^13^C-NMR (DMSO-*d*6), δ, ppm: 18.96 (CH3), 26.27 (CH2), 39.46–40.09 (DMSO-*d*6 + CH2), 54.34–58.54 (CH2), arC: [104.62 (CH), 111.20 (CH), 131.02 (CH), 133.75 (CH), 163.09 (CH), 172.28 (CH)]; UV: 246 nm (8155.26 mol^−1^Lcm^−1^), 283 nm (29869.29 mol^−1^Lcm^−1^). MS: calc. *m/z* 263.34, found 264.17 (M+H).

### 3.5. Anti-AChE Assay

The anti-cholinesterase activities of the compounds were evaluated by Ellmann’s method with slight modifications, using acetylthiocholine as a substrate [29] and 5,5’-dithiobis[2-nitrobenzoic acid](DTNB). Sodium phosphate buffer (pH 8.0, 110 μL) was added into the 96 wells followed by sample solution (20 μL), DTNB (0.126 mM, 50 μL) and AChE enzyme (0.6 U/mL, 20 μL). The mixture was incubated for 50 minutes at 37 °C. The reaction was then initiated by the addition of acetylthiocholine iodide (0.120 mM, 50 μL). The hydrolysis of acetylthiocholine was monitored by the formation of yellow 5-thio-2-nitrobenzoate anion as the result of the reaction of DTNB with thiocholine, released by the enzymatic hydrolysis of acetylthiocholine, at a wavelength of 412 nm every 30 s for 25 min using a 96-well microplate plate reader (TECAN Infinite M200, Mannedorf, Switzerland). Test compounds were dissolved in analytical grade DMSO. Tacrine and propidium iodide were used as reference standards [30]. The reactions were performed in triplicate and monitored with a spectrophotometer. The percent inhibition of the enzyme activity due to the presence of increasing test compound concentration was obtained from the expression; 100 − (*v_i_/v_o_* × *100*), where *v_i_* is the initial rate calculated in the presence of inhibitors and *v_o_* is the enzyme activity.

### 3.6. Molecular Modeling Evaluations

The pdb structure of human acetylcholinesterase (hAChE) (pdb ID: 1B41) was obtained from the Protein Data Bank. Hydrogen atoms were added to the structure, and all ionizable residues were set at their default protonation state at a neutral pH. The 3D structure of the ligand was constructed using ChemBio Office 2008 and optimized according to the standard protocol in Accelrys Discovery Studio 2.1. Docking studies were then carried using CDOCKER protocol at the active site of hAChE based on the Binding-Site module. CDOCKER is a grid based molecular docking method that employs CHARMm. The receptor is held rigid while the ligands are allowed to flex during the refinement. Random ligand conformations are generated from the initial ligand structure through high temperature molecular dynamics, followed by random rotations followed by refinement by grid-based (GRID 1) simulated annealing and a final grid-based or full force field minimization. In this experiment, the ligand was heated to a temperature of 700 K in 2,000 steps. The cooling steps were set to 5,000 steps with 300 K cooling temperature. The grid extension was set to 8 Å and ten conformations were set for the ligand. The pose with the highest –CDOCKER energy was taken for further analysis [31].

### 3.7. Antioxidant Activity

#### 3.7.1. FRAP Assay

The FRAP assay of the compounds performed using modified method as described by Benzie and Strain [32]. The stock solutions contained 300 mM acetate buffer (3.1 g C_2_H_3_NaO_2_·3H_2_O and 16 mL C_2_H_4_O_2_), pH 3.6, 10 mM TPTZ (2,4,6-tripyridyl-*s*-triazine) solution in 40 mM hydrochloric acid and 20 mM ferric chloride hexahydrate solution. The fresh working solution was prepared by mixing acetate buffer (25 mL), TPTZ (2.5 mL), and ferric chloride hexahydrate solution (2.5 mL). The temperature of the solution was raised to 37 °C before use and allowed to react with the FRAP solution (300 μL) in the dark. The colored product (ferrous tripyridyltriazine complex) was monitored at a wavelength of 593 nm. The standard curve was linear between 100 and 1,000 μM ferrous sulphate. Results are expressed in μM ferrous/g dry mass and compared with that of ascorbic acid and butylated hydroxytoluene.

#### 3.7.2. DPPH (1,1-Diphenyl-2-picrylhydrazyl) Assay

The scavenging activities of the compounds on DPPH were recorded according to a reported procedure [33]. The compounds showed final concentrations within the range of 0–25 µg/mL in methanol. One milliliter of 0.3 mM DPPH ethanol solution was added to sample solution (2.5 mL) of different concentrations and used as stock solutions for the test; meanwhile methanol (1 mL) was added to samples (2.5 mL) to make the blank solutions. The negative control (blank) consisted of DPPH solution (1 mL) plus methanol (2.5 mL). These solutions were allowed to react at room temperature for 30 min in the dark. The absorbance was read at 518 nm and converted into percentage antioxidant activity according to the following equation: % Inhibition = [(A_B_ − A_A_)/A_B_] × 100. Where: A_B_: absorption of blank sample, A_A_: absorption of tested samples. The kinetics of DPPH scavenging activity was determined and the IC_50_ calculated using ascorbic acid as a positive control.

### 3.8. Acute Toxicity

The acute toxicity study was carried out using seventy healthy Sprague Dawley rats of either sex weighing between 120–150 g. All animals were obtained from the Experimental Animal House, Faculty of Medicine, University of Malaya, and were divided into seven groups of ten rats each (five males and five females for each group) labeled as control/vehicle (distilled); high dose (5 g/kg) and low dose (2 g/kg) of each of the compounds **LP**, **2HP** and **DHP** in vehicle. Animals were fasted overnight (food but not water) prior to dosing. Food was withheld for a further 3 to 4 h after dosing. The animals were observed for general behavioral changes and mortality every four hours for the period of two weeks and sacrificed on the 15th day. Serum biochemical parameters were determined following standard methods [34]. The experimental animals received human care according to the criteria outlined in the *Guide for the Care and Use of Laboratory Animals* prepared by the National Academy of Sciences and published by the National Institutes of Health [35]. The biochemical analysis was performed at the University Malaya Medical Center laboratory. The blood was centrifuged at 4,000 rpm for 10 min, serum separated and stored at −20 °C until determination of aspartate amino transferase (AST), alanine amino transferase (ALT), total cholesterol (TC), low density lipoprotein (LDL), high density lipoprotein (HDL), triglyceride (TG), alkaline phosphatase (ALP), total bilirubin (TB), conjugated bilirubin (CB), total protein (TP), globulins and albumin (ALB). Renal function indices, such as the concentration of creatinine, urea, sodium, chloride, potassium and inorganic phosphorus were also analyzed. Ethical approval was obtained from the Experimental Scientific Committee, Faculty of Medicine, University of Malaya.

### 3.9. Statistical Analysis

Statistical analysis was carried out using SPSS software version 19.0 and the results expressed as mean ± S.E.M. of the calculated value in each experiment using a one-way analysis of variance, followed by least significance difference (LSD) test for multiple comparisons.

## 4. Conclusions

The novel Schiff bases potentially useful for cholinesterase inhibition were synthesized and characterized. The compounds were found to be safe in animal tests and showed good to moderate AChE inhibition activities. The hydrophobic interaction between the Schiff base **DHP** and the rich aromatic contents of the enzyme along the gorge directed the phenyl ring to penetrate deeply into the anionic site and oxyanion hole regions in the choline-binding site. This interaction is further intensified, whereby three hydrogen bonding were found between the Schiff base **DHP** and the residues in the active site, as confirmed by molecular docking. The antioxidant study indicated fairly good activities in the FRAP assay for all the compounds, while in the DPPH assay only the compound **DHP** showed activity.

## Figures and Tables

**Table 1:** Human AChE inhibitory effects and anti-oxidant activities of **LP**, **2HP** and **DHP**.

Compounds	Molecular Weight	AChE Inhibition (Final concentration = 1 × 10^−4^ M)	DPPH (IC_50_, µg/mL)	FRAP value (Mean ± SD)
**LP**	232.3	9.9 ± 3.1	-	1464.7 ± 5.2
**2HP**	247.3	−4.0	-	293.3 ± 6.2
**DHP**	263.3	21.8 ± 1.2	25 ± 1.24	355.3 ± 5.8
Propidium	-	28.2 ± 2.4	-	-
Tacrine	-	76.6 ± 5.1	-	-
Ascorbic acid	-	-	1.4 ± 0.71	1552.7 ± 4.2
BHT	-	-	NA	187.3 ± 2.6

**LP**: Schiff base derived from 2-acetylpyridine, **2HP**: Schiff base derived from 2-hydroxy-acetophenone; **DHP**: Schiff base derived from dihydroxyacetophenone; BHT: Butylated hydroxytoluene (standard positive antioxidant); NA: not applicable.

**Table 2:** Renal function test of rats used in acute toxicity assessments of 1-(2-ketoiminoethyl)piperazine Schiff bases.

Animals	Sodium (mmol/L)	Potassium (mmol/L)	Chloride (mmol/L)	Carbon dioxide (mmol/L)	Anion gap (mmol/L)	Urea (mmol/L)	Creatinine (µmol/L)
Control	132.9 ± 2.1	6.14 ± 2.5	111.30 ± 3.5	16.12 ± 2.6	32.80 ± 2.9	7.40 ± 2.5	29.45 ± 9.6
2HP (2 g/kg)	132.2 ± 3.5	6.27 ± 4.1	108.24 ± 4.2	19.18 ± 0.8	29.60 ± 1.2	7.10 ± 4.2	27.60 ± 6.4
2HP (5 g/kg)	133.1 ± 4.1	6.35 ± 3.2	106.31 ± 3.8	17.51 ± 2.7	29.50 ± 3.6	7.30 ± 3.7	25.91 ± 8.3
DHP (2 g/kg)	133.5 ± 0.9	6.29 ± 0.4	109.71 ± 6.2	18.31 ± 0.4	28.70 ± 4.2	6.90 ± 2.1	26.22 ± 2.1
DHP (5 g/kg)	134.1 ± 1.8	6.37 ± 0.7	108.19 ± 2.4	19.72 ± 0.8	30.40 ± 3.2	7.10 ± 1.4	28.47 ± 1.6
LP (2 g/kg)	131.3 ± 2.2	6.52 ± 0.9	110.32 ± 2.1	18.34 ± 1.3	31.20 ± 2.5	7.01 ± 0.9	27.90 ± 1.3
LP (5 g/kg)	133.1 ± 0.4	6.44 ± 1.2	107.82 ± 3.2	14.26 ± 1.5	33.10 ± 3.4	6.8 0± 2.1	30.13 ± 0.4

Values expressed as mean ± S.E.M. There are no significant differences between groups. Significant value at p < 0.05.

**Table 3:** Liver function test of rats used in acute toxicity assessments of 1-(2-ketoiminoethyl)piperazine Schiff bases.

Animals	Total protein (g/L)	Albumin (g/L)	Globulin (g/L)	TB (µmol/L)	CB (µmol/L)	AP (IU/L)	ALT (IU/L)	AST (IU/L)
Control	64.10 ± 2.2	18.40 ± 1.1	50.90 ± 2.3	6.20 ± 0.2	3.20 ± 0.6	104.20 ± 12.4	56.20 ± 4.9	220.12 ± 4.9
2HP (2 g/kg)	65.00 ± 2.7	18.20 ± 1.2	51.60 ± 2.1	8.30 ± 0.3	4.30 ± 0.2	110.10 ± 11.3	62.12 ± 4.4	236.23 ± 6.1
2HP (5 g/kg)	66.12 ± 2.2	19.20 ± 1.1	49.90 ± 2.5	9.50 ± 0.6	5.20 ± 0.4	101.50 ± 10.8	6515 ± 4.6	232.18 ± 4.3
DHP (2 g/kg)	64.21 ± 2.6	20.10 ± 1.2	52.10 ± 2.5	8.90 ± 0.6	6.40 ± 0.5	106.40 ± 11.2	71.15 ± 3.8	235.21 ± 4.7
DHP (5 g/kg)	66.16 ± 2.1	20.30 ± 1.3	54.20 ± 2.6	9.10 ± 0.8	6.60 ± 0.4	115.30 ± 10.8	70.67 ± 4.3	242.16 ± 5.1
LP (2 g/kg)	65.32 ± 2.4	21.20 ± 1.8	50.10 ± 2.5	7.90 ± 0.9	7.10 ± 0.8	118.20 ± 10.5	69.45 ± 4.4	239.31 ± 4.8
LP (5 g/kg)	63.23 ± 2.3	18.40 ± 1.6	52.20 ± 2.6	8.80 ± 0.4	7.20 ± 0.8	114.50 ± 10.1	68.35 ± 4.1	234.17 ± 4.6

Values expressed as Mean ± S.E.M. There are no significant differences between groups. Significant value at p < 0.05 TB: Total bilirubin; CB: Conjugated bilirubin; AP: Alkaline phosphatase; ALT: Alanine aminotransferase; AST: Aspartate aminotransferase.
